# Polymorphism and expression of *GLUD1* in relation to reproductive performance in Jining Grey goats

**DOI:** 10.5194/aab-66-411-2023

**Published:** 2023-12-07

**Authors:** Wei Wang, Yongjuan Wang, Yufang Liu, Guiling Cao, Ran Di, Jinyu Wang, Mingxing Chu

**Affiliations:** 1 Key Laboratory of Animal Genetics, Breeding and Reproduction of Ministry of Agriculture and Rural Affairs, Institute of Animal Science, Chinese Academy of Agricultural Sciences, Beijing 100193, China; 2 College of Animal Science and Technology, Yangzhou University, Yangzhou 225009, China

## Abstract

Understanding the molecular mechanism of mammalian reproduction (puberty and prolificacy) will play a part in improving animal reproductive performance. *GLUD1* (glutamate dehydrogenase 1) is important for mammalian reproduction, as shown in previous studies; however, its roles in puberty and prolificacy have rarely been reported. In this study, we designed seven pairs of primers (P1 to P7) for cloning and sequencing genomic DNA of Jining Grey goats and Liaoning Cashmere goats. Primer 8 (P8) was designed to detect single nucleotide polymorphism (SNP) of the *GLUD1* in both sexually precocious and high-fecundity breeds (Jining Grey, Nanjiang Brown and Matou goats) and sexually late-maturing and low-fecundity breeds (Liaoning Cashmere, Inner Mongolia Cashmere and Taihang goats) by PCR-RFLP (restriction fragment length polymorphism). The real-time quantitative polymerase chain reaction (RT-qPCR) technique was used to detect the expression of *GLUD1* in a variety of tissues. The results showed that the A197C mutation was only found in the amplification product of P6. For this SNP locus, only two genotypes (AA and AC) were detected in Nanjiang Brown goats, while three genotypes (AA, AC and CC) were detected in the other five breeds. In Jining Grey goats, the frequency of genotypes AA, AC and CC was 0.69, 0.26 and 0.05, respectively. In Jining Grey goats, AA genotype had 0.54 ( P<0.05 ) and 0.3 ( P<0.05 ) more kids than the CC and AC genotype, respectively, and no significant difference ( P>0.05 ) was found in kidding number between the AC and CC genotype. *GLUD1* was expressed in five tissues of different developmental stages. The expression level of *GLUD1* in the hypothalamus was higher than that in the other four tissues except during puberty of Liaoning Cashmere goats. In puberty in goats, *GLUD1* expression was significantly higher in ovaries than that in the juvenile period ( P<0.01 ). RT-qPCR results showed that the expression of *GLUD1* in ovaries may relate to the puberty of goats. The present study preliminarily indicated that there might be an association between the 197 locus of *GLUD1* and sexual precocity in goats, and allele A of *GLUD1* was a potential DNA marker for improving kidding number in Jining Grey goats.

## 1 Introduction

Glutamate is the dominant excitatory amino acid neurotransmitter in neuroendocrine regulation. The availability of releasable glutamate from neurochemicals promotes a rapid increase in gonadotropin-releasing hormone (GnRH) secretion and enhanced GnRH mRNA and protein content, which was essential for controlling the onset of puberty (Van Den Pol et al., 1990; Brann and Mahesh, 1997; Terasawa and Fernandez, 2001). Previous studies revealed that the expression levels of glutamate were extremely low in pre-puberty; increased markedly in early puberty; and then maintained higher levels in puberty, which were associated with the expression of hypothalamic glutamate dehydrogenase (GDH) (Erecińska and Silver, 1990; Bourguignon et al., 1995; Terasawa et al., 1999; Roth et al., 2006). Significant alteration in the glutamate dehydrogenase activities may decrease the metabolism of glutamate, leading to the accumulation of extracellular glutamate (Malthankar-Phatak et al., 2006). Both glutamate and GDH were predominantly expressed in glial cells, suggesting that glial-cell-derived GDH output played a major role in the regulation of GnRH release in puberty (Terasawa and Fernandez, 2001). *GLUD1* (glutamate dehydrogenase 1) is one of the enzymes which acts in the process of ammonia assimilation and is a hexameric enzyme with wide distribution. Some studies indicated that hormonal changes at puberty always had an extremely large impact on seizures and epilepsy (Mmako and Magazi, 2017; Odintsova et al., 2017; van Luijtelaar et al., 2017; Stefanidou and Montouris, 2020). Epilepsy onset was prevalent at the stages of the beginning of estradiol level increase and its ovulatory peaks (Odintsova et al., 2017; van Luijtelaar et al., 2017), suggesting that *GLUD1* might play a role in precocious puberty.

Regulated by multiple endogenous, genetic and environmental factors, puberty is a complex and dynamic period in the development of goats, which is characterized by sexual maturation and the onset of reproductive capability. Puberty is controlled by glial cells, which regulate the availability of glutamate to the neuronal-glial network controlling the gonadotropin-releasing hormone (GnRH) (Roth et al., 2006). Puberty is the period of transition between infertility and fertility. Speeding up puberty transition would greatly reduce the production cost of reserve goats. In humans, shortened puberty is a sign of precocious puberty, which is mostly pathological (Sultan et al., 2018). However, some animal species have evolved precocious traits that can be stably inherited. Therefore, early sexual maturity may be an important quantitative trait in animal production. Genes associated with precocious puberty have attracted the attention of many scientists; ongoing research is likely to find out the main role of genes or closely linked molecular markers, effectively used to provide strong theoretical support for the prevention of human sexual premature disease and the breeding of animal varieties of early maturity and high fecundity (Maitra et al., 2014). During late postnatal development, GnRH in the hypothalamus drives the synthesis and secretion of follicle-stimulating hormone (FSH) and luteinizing hormone (LH), which are secreted through the hypothalamic–pituitary–ovarian axis and stimulate the gonads to trigger puberty. The secretion of GnRH neurons depended on various factors, such as γ -aminobutyric acid (GABA), G-protein-coupled receptor 54 (GPR54) and glutamate (Iremonger et al., 2010; Pineda et al., 2010; Watanabe et al., 2014). The kidding number is one of the most important economic traits in goats. Research on key genes of kidding can provide useful information for marker-assisted breeding to improve kidding number. Previous studies have proved that gene polymorphism can affect the reproductive performance of goat (Maitra et al., 2014; Tao et al., 2020).

Precocious puberty refers to the premature appearance of secondary sexual characteristics in individual goats, while delayed puberty is the opposite. Nanjiang Brown goats, Matou goats and Jining Grey goats are highly prolific local breeds in PR China. Taihang goats, Inner Mongolia Cashmere goats and Liaoning Cashmere goats are lowly prolific local breeds in PR China. Jining Grey goats are precocious and generally reach estrus at the age of 2–3 months (Tu, 1989); Nanjiang Brown goats are precocious and begin estrus at the age of 4 months (Du, 2011); Matou goats mature earlier, and the average age of estrus of goats is 107.9 d (Tu, 1989); Liaoning Cashmere goats mature late and reach estrus at the age of 5–6 months (Tu, 1989); Inner Mongolia Cashmere goats mature late and reach estrus at the age of 5–6 months (Tu, 1989); and Taihang goats generally reach estrus at 6–7 months of age (Tu, 1989).

In this study, *GLUD1* single nucleotide polymorphisms (SNPs) were analyzed in early-puberty goats (Nanjiang Brown goats, Matou goats and Jining Grey goats) and delayed-puberty goat breeds (Taihang goats, Inner Mongolia Cashmere goats and Liaoning Cashmere goats) by sequencing and restriction fragment length polymorphism (RFLP). The real-time quantitative polymerase chain reaction (RT-qPCR) technique was used to study the expression of *GLUD1* in different tissues of the hypothalamus, pituitary gland, ovaries, uterus and adrenal gland of Jining Grey goats and Liaoning Cashmere goats at 30 ± 5, 90 ± 5 and 160–180 d. The present study aimed to find genetic markers related to sexual precocity and high fertility in goats and to provide a scientific basis for marker-assisted early selection of reproductive performance.

## 2 Material and methods

### 2.1 Animal preparation and sample collection

Jugular blood samples from 475 does (2–3 years) were processed with phenol–chloroform and dissolved in ddH ${}_{2}$ O. Among the six goat breeds involved in the present study, there were three precocious-puberty goat breeds (Nanjiang Brown goats, n=30 ; Matou goats, n=48 ; Jining Grey goats, n=212 ) and three delayed-puberty goat breeds (Taihang goats, n=55 ; Inner Mongolia Cashmere goats, n=59 ; Liaoning Cashmere goats, n=71 ) (Table 1, Supplement Fig. S1) (Du, 2011; Feng et al., 2011). Five tissue samples including the hypothalamus, pituitary gland, ovaries, uterus and adrenal gland were taken from Jining Grey goats and Liaoning Cashmere goats. All the tissue samples were collected within 1 h after the goats were slaughtered and preserved in a 2 mL RNase-Free frozen storage tube in liquid nitrogen. Then, all samples were taken back to the laboratory and stored at - 80 ${}^{\circ }$ C.

The 212 Jining Grey goats were randomly selected as the offspring of five bucks, whose parities, kidding months and litter size were recorded. The first parity ( n=62 ), the second parity ( n=68 ) and the third parity ( n=82 ) of Jining Grey goats were defined as three parities. According to the actual kidding time, March to May was taken as spring kidding ( n=56 ), June to August as summer kidding ( n=47 ), September to November as autumn kidding ( n=65 ) and December to February as winter kidding ( n=44 ).

**Table 1:** Basic information on does used in this study.

Breed	N	Puberty	District	Farms
Nanjiang Brown goat	30	precocious puberty	Bazhong, Sichuan Province, China	Nanjiang Brown Goat Original Breeding Farm
Matou goat	48	precocious puberty	Shiyan, Hubei Province, China	Shiyan City, Hubei Province Goat Farm
Jining Grey goat	212	precocious puberty	Jining, Shandong Province, China	Jining Grey Goat Breeding Base
Taihang goat	55	delayed puberty	Jiaozuo, Henan Province, China	Goat farm in Wuzhi County, Henan Province
Inner Mongolia Cashmere goat	59	delayed puberty	Ordos, Inner Mongolia Autonomous Region, China	Inner Mongolia Cashmere Goat Breeding Farm
Liaoning Cashmere goat	71	delayed puberty	Liaoyang, Liaoning Province, China	Liaoning Cashmere Goat Breeding Center

N : number of individual goats.

### 2.2 RNA extraction and primer design

Total RNA was isolated from samples using the TRIzol reagent (Invitrogen, Carlsbad, CA, USA) according to the manufacturer's instructions. RNA purity was confirmed with a NanoPhotometer spectrophotometer (IMPLEN, CA, USA), and concentration and integrity were assessed using electrophoresis and the RNA 6000 Nano assay kit of the 2100 Bioanalyzer system (Agilent Technologies, Santa Clara, CA, USA).

To amplify the *GLUD1* coding sequence of goats, eight primer pairs were designed according to DNA fragments (contig) containing the region of interest using Primer Premier software (version 5.0) and Oligo software (version 6.0), and synthesized by Sangon Biotech Co. Ltd. (Shanghai, China) (Table 2).

**Table 2:** Primer sequence, amplified region, fragment size and annealing temperature for goat *GLUD1*.

Name	Primer sequence ( $5^{\prime }\to 3^{\prime }$ )	Amplified	Product	Annealing temperature
		region	size (bp)	( ${}^{\circ }$ C)
P1	F: TGGGTTCCGTGGCTGCCGACT	Exon 1	597	67
	R: CTCGCGGGGGGAGGACCAACT			
P2	F: CCAGGTATCCGTTACAGCACT	Exon 2	137	56
	R: CCAAACATTTTCCCAGAGTTCT			
P3	F: GAATAACAGCTTTCAAACTTCT	Exon 3	117	53
	R: ACCATGTGCACTAATGTCAT			
P4	F: AGTCTCTTGAGTGGTGTATCTGT	Exon 5	349	47.5
	R: CTATGGTGCTGGCGTAGGT			
P5	F: CCTGCACTCTATGAGATATT	Exon 7	138	49
	R: CTATTAGAACAACATACCAATT			
P6	F: TCATATCATAGGCACTGAACAT	Exon 8–exon 9	649	55
	R: TCACTAAGGCTTGGGAAACT			
P7	F: CGGTAGAACAAGTTTGGAATAAG	Exon 12	434	57
	R: ATGACCCTGGAAACAACACT			
P8	F: AAGGGAGCATCTTGGAGGTC	Exon 8 and intron 8	382	60.5
	R: GGAGTTGTTGGTCCGTTGG			

### 2.3 PCR amplification and real-time quantitative PCR

The PCR amplification reaction system is shown in Table 3. The reaction program is as follows: 95 ${}^{\circ }$ C predenaturation for 5 min, 95 ${}^{\circ }$ C denaturation for 30 s, annealing for 30 s and 72 ${}^{\circ }$ C extension for 30 s for 35 cycles and then 72 ${}^{\circ }$ C extension for 5 min. After PCR, the product was stored at 4 ${}^{\circ }$ C.

The experimental process of RT-qPCR was the same as described by Hu et al. (2020) in our laboratory. In short, cDNA was synthesized according to the steps provided by the reverse transcription kit; 2 × *Taq* PCR Master Mix was used to perform PCR on *GLUD1*, and SYBR^®^Premix Ex Taq™ II was finally used for RT-qPCR. The experiment was carried out using the Roche Light Cycler^®^480 II fluorescent quantitative PCR instrument, with *GAPDH* as the internal reference gene, and this was repeated for each sample three times. The 2 ${}^{-\Delta \Delta ct}$ method (Hu et al., 2021) was used to calculate the relative expression of the target gene. SPSS 24.0 statistical software was used to perform a one-way analysis of variance (ANOVA) on the data, and the least significant difference (LSD) test was used to evaluate differences between groups. The P<0.05 value was considered to indicate significance.

### 2.4 Cloning and sequencing

Following the instructions of the manufacturer, the amplified fragments were separated on agarose gels (1.5 %) and purified with a DNA fragment quick recover kit (TaKaRa, Dalian, China), and they were then inserted into the pMD18-T vector (TaKaRa, Dalian, China). The recombinant plasmids were transformed into *E.coli* JMl09 competent cells. Positive clones were identified by restriction enzyme digestion and sequenced by Beijing Genomics Institute (China). To identify different nucleotides, DNAMAN (v. 5.2.2) was used for sequence analysis.

**Figure 1:**
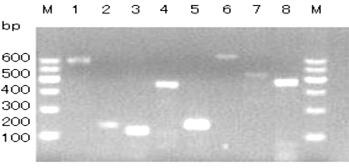
Agarose gel electrophoresis of PCR products of primers P1–P8. Bands 1–8 are products of P1–P8, respectively. M: DNA Marker I.

### 2.5 PCR-RFLP analysis

The restriction enzyme digestion mixture contained 1 µ L 10 × NEB buffer, 0.3 µ L restriction endonuclease *Bso*BI (10 U µ L ${}^{-1}$ , NEB), 4 µ L PCR product (P8 primer) and 4.7 µ L ddH ${}_{2}$ O. The reaction was held at 37 ${}^{\circ }$ C for 5 h. The digested products were analyzed by electrophoresis on 12 % polyacrylamide gel (acrylamide : bis-acrylamide = 39:1 ). AlphaImager™ 2200 and 1220 Documentation and Analysis Systems (Alpha Innotech Corporation, San Leandro, CA, USA) were used to analyze and count genotypes.

### 2.6 Statistical analyses

Allele frequencies, genotype frequencies, p values, polymorphism information content (PIC), heterozygosity (HE) and the number of effective alleles (NE) were calculated using the data obtained from genotyping results. The chi-squared test was used to detect the Hardy–Weinberg (H-W) equilibrium of each mutation site in six goat populations (Ortega et al., 2016). We performed least-squares analysis of variance by SAS with the following model to analyze the genotypes of primer P8 and compare the differences in litter size of Jining Grey goats with different genotypes.

1 $$y_{ijklm}=\mu +S_{i}+{\text{KS}}_{j}+P_{k}+G_{l}+e_{ijklm},$$

where $y_{ijklm}$ is the recorded value of litter size, μ is the overall population mean, $S_{i}$ is the fixed effect of the male goat ( i=1 , 2, 3, 4, 5), KS ${}_{j}$ is the fixed effect of the kidding season ( j=1 , 2, 3, 4), $P_{k}$ is the fixed effect of the parity ( k=1 , 2, 3), $G_{l}$ is the fixed effect of the genotype and $e_{ijklm}$ is a random residual effect.

## 3 Results and analysis

### 3.1 PCR amplification

The *GLUD1* of six goat breeds was amplified by primers P1 to P8, and the PCR products were detected by 1.5 % agarose gel electrophoresis. The results showed that the amplified fragments were consistent with the expected size, and specific fragments were obtained (Fig. 1).

**Table 3:** PCR amplification reaction system.

Reagent	Volume
ddH ${}_{2}$ O	11 µ L
10 × PCR buffer (Mg ${}^{2+}$ free)	2.0 µ L
dNTPs (2.5 mM each)	2.0 µ L
MgCl ${}_{2}$ (20 mM)	1.5 µ L
Primer F (10 µ M)	0.5 µ L
Primer R (10 µ M)	0.5 µ L
r*Taq* DNA polymerase (2.5 U µ L ${}^{-1}$ )	0.5 µ L
DNA (50–100 ng µ L ${}^{-1}$ )	2.0 µ L
Total volume	20.0 µ L

### 3.2 Sequence analysis of amplification fragments

**Figure 2:**
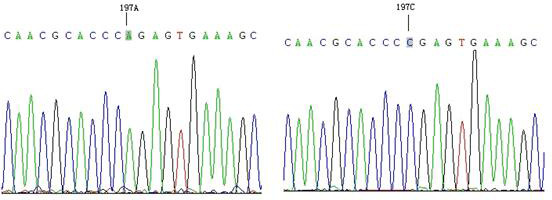
Nucleotide mutation of *GLUD1* in goats.

The results showed that only one mutation site (A197C) was found in the amplified product of P6, and fragment P6 corresponds to exon8–exon9. Compared with the AA genotype, the CC genotype had an A → C mutation at position 197 (Fig. 2), which did not cause amino acid changes and was a silent mutation.

**Figure 3:**
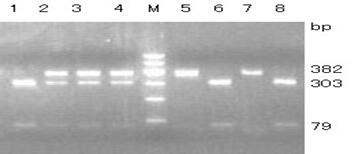
Agarose gel electrophoresis for the RFLP-PCR product of the amplified fragment belonging to exon 8 and intron 8. Lines 5 and 7, AA; lines 2, 3 and 4, AC; lines 1, 6 and 8, CC. M: DNA Marker I (100, 200, 300, 400, 500, 600).

### 3.3 RFLP analysis of the amplified fragment

One pair of new primers P8 was designed to detect the A → C mutation at position 197. The restriction enzyme *Bso*BI was used to directly perform RFLP detection. As shown in Fig. 3, there were three genotypes: AA (382 bp), AC (79/303/382 bp) and CC (79/303 bp).

### 3.4 The expression level of goat *GLUD1* in various tissues

We calculated the RT-qPCR results of the target gene of each sample. We also contrasted the expression difference in mRNA transcription level of the target gene in other samples relative to the reference sample (the average value of the ovaries in the juvenile period of Jining Grey goats was the reference sample). The results are shown in Table 4.

**Table 4:** The expression of *GLUD1* in different tissues of Jining Grey and Liaoning Cashmere goats.

Breed	Age	N	Hypothalamus	Pituitary	Ovary	Uterus	Adrenal gland
Jining Grey goat	Juvenile period I	3	7.61 ${}^{\mathrm{AB}}$	0.59 ${}^{B}$	1.00 ${}^{B}$	1.76 ${}^{B}$	2.10 ${}^{B}$
	(30 ± 5 d)		± 2.94	± 0.06	± 0.17	± 0.18	± 0.78
	Puberty	3	3.46 ${}^{B}$	0.79 ${}^{\mathrm{AB}}$	2.14 ${}^{A}$	2.23 ${}^{B}$	2.04 ${}^{B}$
	(90 ± 5 d)		± 1.34	± 0.28	± 0.10	± 0.17	± 0.64
Liaoning Cashmere goat	Juvenile period I	3	8.61 ${}^{A}$	1.14 ${}^{\mathrm{AB}}$	1.04 ${}^{B}$	2.31 ${}^{B}$	3.39 ${}^{A}$
	(30 ± 5 d)		± 0.72	± 0.37	± 0.16	± 1.07	± 0.94
	Juvenile period I	3	7.64 ${}^{\mathrm{AB}}$	1.48 ${}^{A}$	1.59 ${}^{\mathrm{AB}}$	2.90 ${}^{B}$	2.74 ${}^{B}$
	(90 ± 5 d)		± 1.42	± 0.38	± 0.35	± 0.60	± 0.34
	Puberty	3	5.84 ${}^{\mathrm{AB}}$	0.92 ${}^{\mathrm{AB}}$	2.21 ${}^{A}$	14.28 ${}^{A}$	2.96 ${}^{B}$
	(160–180 d)		± 0.65	± 0.31	± 0.41	± 2.24	± 1.22

The values are averages. In the same column, different uppercase letters next to means indicate differences ( P<0.01 ), and the same letters indicate nonsignificant differences. In the same row, different uppercase letters next to means indicate differences ( P<0.01 ), and the same letters indicate nonsignificant differences. N : number of individual goats.

*GLUD1* was expressed in five tissues at each developmental stage of Jining Grey goats and Liaoning Cashmere goats (Table 4). The results showed that the expression of *GLUD1* in the hypothalamus of high-fecundity breeds (Jining Grey goats) was significantly higher than that in the other four tissues ( P<0.01 ), and it was the lowest in the pituitary. The expression level of *GLUD1* in the hypothalamus in the juvenile period I of Jining Grey goats was significantly higher than that in puberty ( P<0.0 1). There were no significant differences between the expression levels of *GLUD1* in the other three tissues of the Jining Grey goats in different periods. In the low-fecundity breed (Liaoning Cashmere goats), the expression of *GLUD1* in the hypothalamus in the juvenile period (I and II) was significantly higher than that in the other four tissues ( P<0.01 ). However, the expression of *GLUD1* in the hypothalamus and uterus at puberty was significantly higher than that of the other three tissues ( P<0.01 ). Except for the juvenile stage I of Liaoning Cashmere goats, the expression of *GLUD1* was the lowest in the pituitary at all stages ( P<0.01 ).

In juvenile period I, the expression level of *GLUD1* in the hypothalamus of Liaoning Cashmere goats was significantly higher than that in Jining Grey goats ( P<0.01 ). During puberty, the expression levels of *GLUD1* in the uterus of Liaoning Cashmere goats were lower than those in Jining Grey goats ( P<0.01 ).

### 3.5 Allele and genotype frequencies of *GLUD1* in six goat breeds

The allele and genotype frequencies of the *GLUD1* mutation site in six goat breeds were shown in Table 5. The results showed that the A allele was the major allele in the five goat breeds, while only the C allele in the Liaoning Cashmere goat was the dominant allele. All populations were in a Hardy–Weinberg equilibrium state ( P>0.05 ).

**Table 5:** Allele and genotype frequencies of the SNP in *GLUD1* in six goat breeds.

Breed	N	Genotype frequency			Allele frequency		H-W test	PIC	HE	NE
		AA	AC	CC	A	C	$\chi^{2}$			
Jining Grey goat	212	0.69(146)	0.26(56)	0.05(10)	0.82	0.18	2.22	0.25	0.3	1.42
Nanjiang Brown goat	30	0.80(24)	0.20(6)	0(0)	0.9	0.1	0.37	0.16	0.18	1.22
Matou goat	48	0.67(32)	0.25(12)	0.08(4)	0.79	0.21	2.81	0.27	0.33	1.48
Liaoning Cashmere goat	71	0.20(14)	0.58(41)	0.22(16)	0.49	0.51	1.72	0.37	0.5	2
Inner Mongolia Cashmere goat	59	0.51(30)	0.47(28)	0.02(1)	0.75	0.25	3.73	0.31	0.38	1.61
Taihang goat	55	0.54(30)	0.44(24)	0.02(1)	0.76	0.24	2.4	0.3	0.36	1.57

The numbers in parentheses are the number of individuals corresponding to the genotype. PIC: polymorphism information content. HE: heterozygosity. NE: effective allele numbers. N : number of individual goats.

### 3.6 Genotype distribution of *GLUD1* in six goat breeds

The differences in the distribution of different genotypes at the A197C locus among the six goat breeds were tested (Table 6). The results showed that there was a significant difference in the distribution of genotypes at this locus between the high-fecundity breed (Jining Grey goats) and the low-fecundity breeds (Liaoning Cashmere goats, Inner Mongolia Cashmere goats and Taihang goats) ( P<0.01 ). Besides, another significant difference was found between the other high-fecundity breeds (Nanjiang Brown goats and Matou goats) and the low-fecundity breed (Liaoning Cashmere goats) ( P<0.01 ). There was also a significant difference between the high-fecundity breeds (Nanjiang Brown goats and Matou goats) and the low-fecundity breed (Inner Mongolia Cashmere goats) ( P<0.05 ). These results indicated that this locus might be associated with the litter size of goats.

**Table 6:** Test of difference of genotype distributions of the 197 locus of *GLUD1* gene in six goat breeds.

Breed	Jining	Nanjiang	Matou	Liaoning	Inner Mongolia	Taihang
	Grey goat	Brown goat	goat	Cashmere goat	Cashmere goat	goat
Jining Grey goat		2.30	1.01	56.34 ${}^{**}$	9.94 ${}^{**}$	6.57 ${}^{*}$
Nanjiang Brown goat			3.16	33.59 ${}^{**}$	7.22 ${}^{*}$	5.60
Matou goat				26.66 ${}^{**}$	7.21 ${}^{*}$	5.41
Liaoning Cashmere goat					20.57 ${}^{**}$	21.82 ${}^{**}$
Inner Mongolia Cashmere goat						0.17
Taihang goat						

${}^{*}$ P<0.05 . ${}^{**}$ P<0.01 . The numbers in the table are the rates of similarity in genotype distribution among different breeds.

### 3.7 The effect of *GLUD1* on litter size of Jining Grey goats

The average litter size and standard errors of different genotypes of *GLUD1* in Jining Grey goats are shown in Table 7. For the 197 locus, the average litter size of AA type Jining Grey goats had 0.54 ( P<0.05 ) and 0.30 ( P<0.05 ) kids more than that of CC- and AC-type Jining Grey goats, respectively. There was no significant difference in litter size between AC and CC types.

**Table 7:** Least-squares mean and standard error for litter size of different genotypes of the 197 locus of *GLUD1* in Jining Grey goats.

Locus	Genotype	Number of does	Litter size
197	AA	146	2.44 ${}^{a}$ ± 0.07
	AC	56	2.14 ${}^{b}$ ± 0.11
	CC	10	1.90 ${}^{b}$ ± 0.17

Least-squares means with the same superscript have no significant difference ( P>0.05 ). The least-squares means with the different superscripts differ significantly ( P<0.05 ).

## 4 Discussion

### 4.1 Expression of *GLUD1*

In recent studies, *GLUD1* mainly functioned as an amino-acid-metabolism-related gene, and cytoplasmic *GLUD1* degradation was shown to limit protein synthesis (Shao et al., 2021). In *Saccharomyces cerevisiae*, glutamate can be synthesized from α -ketoglutarate and ammonium through the action of the NADP-dependent glutamate dehydrogenases *GLUD1* and GDH3 (Mara et al., 2018). However, the role played by the expression of glutamate dehydrogenase in pubertal development cannot be ignored. It has been found that the expression of glutamate dehydrogenase in the hypothalamus of female rats increased during the development of puberty, while the expression of glutamate synthetase catalyzed the metabolism of glutamate from glutamine (Hasan Mahmood et al., 2018; Ibrahim et al., 2020; Uddin et al., 2020). Spanaki et al. (2021) mentioned that *GLUD1* was intensively expressed in estrogen-producing cells in human ovaries and placenta, and it was a housekeeping enzyme (Spanaki et al., 2017), which was also reflected in our study. The results of our study showed that *GLUD1* was highly expressed in ovaries of Jining Grey goats and Liaoning Cashmere goats at various developmental stages, and the expression in the first estrus was higher than that in juvenile period. It was speculated that the change of *GLUD1* expression in ovaries affected the first estrus. In human steroidogenic organs, *GLUD1* was highly expressed in steroid synthesizing cells with this expression pattern, matching that of the cholesterol side-chain system involved in steroid biosynthesis. However, there were few studies focused on the expression of *GLUD1* in goats. The expression of *GLUD1* in five tissues of goats was analyzed in this study, and there was no significant difference in the expression of *GLUD1* in the hypothalamus between Jining Grey goats and Liaoning Cashmere goats in the same period. Except for Liaoning Cashmere goats at the first stage of estrus, the expression levels in the hypothalamus were shown to be higher than those in the other four tissues in all other stages.

### 4.2 Polymorphism and function of *GLUD1*

In this study, goat *GLUD1* had a mutation site of A197C in the exon 8 region, which did not alter the amino acid sequence and was a silent mutation. This study also showed that there were obvious differences between sexually early-maturing breeds and late-maturing breeds in the 197 mutation site of different genotypes. Therefore, it was inferred that A197C of *GLUD1* may have an association with the precocious puberty of goats. At present, plenty of studies have focused on the litter size in livestock (Tao et al., 2020, 2021). Previous studies showed that polymorphisms of *BMPR1B* could affect the sheep litter size and that *BMPR1B* affects ovulation in mammals by promoting follicular development and ovarian granulosa cell proliferation (Hu et al., 2020; Wen et al., 2021). However, few studies associating the *GLUD1* polymorphisms with litter size have been published. It was proved that gene polymorphism can affect the number of kidding, which was consistent with previous studies. In previous studies, *GLUD1* was found to contribute to glucose-stimulated insulin secretion in mouse beta cells (Lin et al., 2012; Goldsmith et al., 2014), indicating that its regulation of autophagy was sensitive to the metabolic environment. *GLUD1* could reduce NAD ${}^{+}$ and/or NADP ${}^{+}$ to NADH and/or NADPH, which promoted biological processes such as ammonia handling and Krebs cycle anaplerosis by linking amino acids to carbohydrate metabolism (Spanaki et al., 2017). Moreover, mammalian GDH (*hGLUD1* in humans) has acquired an energy-sensing mechanism (GTP–ADP binding) that regulated enzyme function according to cellular energy needs (Mastorodemos et al., 2005). However, the mechanism of *GLUD1* in goat reproduction has not been clarified. Our study preliminarily indicated that allele A at locus 197 of the *GLUD1* might increase the kidding number in Jining Grey goats, which might be a potentially effective DNA marker for increasing kidding of goats. The samples of Jining Grey goats in this study were all from the same breeding farm, so there might be some kinship between individuals, and the effective population size of the tested goats was reduced, which affected the degree of association between *GLUD1* and kidding number. This also confirms the previous findings that genetic polymorphisms can affect kidding numbers in goats and that studying the role of *GLUD1* polymorphisms can provide useful DNA markers for selecting quality individuals by marker-assisted selection (MAS) and contribute to goat breeding (Yang et al., 2021). In the next step, we will increase the number of goat breeds, expand the number of samples within the breed and conduct further studies.

## 5 Animal ethics

All the experimental procedures were authorized by the Science Research Department of the Institute of Animal Sciences, Chinese Academy of Agricultural Sciences (IAS-CAAS; Beijing, China). The study has also been ethically approved by the Animal Ethics Committee of the IAS (IAS2020-62).

## 6 Conclusions

In this study, it was found that *GLUD1* was expressed in five tissues at all developmental stages in goats. Except for the puberty of Liaoning Cashmere goat, the expression in the hypothalamus at other stages was higher than that in the other four tissues. In ovaries, the expression of *GLUD1* in puberty was significantly higher than that in infancy, which speculated that the change of *GLUD1* expression in ovaries affected puberty. The difference test of our research also showed that *GLUD1* might affect puberty of goats. A mutation at position 197 of *GLUD1* has been detected, and association analysis showed that allele A of this locus is associated with an increase in the number of kidding in Jining Grey goats.

## Supplement

10.5194/aab-66-411-2023-supplement The supplement related to this article is available online at: https://doi.org/10.5194/aab-66-411-2023-supplement.

## Data Availability

No data sets were used in this article.
