# Supplementary material for: Polymorphism and expression of GLUD1 in relation to reproductive performance in Jining Grey goats
Source: Arch Anim Breed. 2023 Dec 7;66(4):411–9. doi: 10.5194/aab-66-411-2023 (PMC10776882; doi:10.5194/aab-66-411-2023)
Supplement: The supplement related to this article is available online at: https://doi.org/10.5194/aab-66-411-2023-supplement. [file aab-66-411-supplement.zip › aab-66-411-2023-supplement-title-page.pdf]

Supplement of Arch. Anim. Breed., 66, 411–419, 2023
https://doi.org/10.5194/aab-66-411-2023-supplement
© Author(s) 2023. CC BY 4.0 License.

Open Access
Archives Animal Breeding

Supplement of

Polymorphism and expression of *GLUD1* in relation to reproductive performance in Jining Grey goats

Wei Wang et al.

Correspondence to: Jinyu Wang (jywang@yzu.edu.cn) and Mingxing Chu (mxchu@263.net)

- aab-66-411-2023-supplement-title-page.pdf
- Figure S1. Photos for different breeds.jpg

The copyright of individual parts of the supplement might differ from the article licence.
