# Supplementary figures and images for: Polymorphism and expression of GLUD1 in relation to reproductive performance in Jining Grey goats
Source: Arch Anim Breed. 2023 Dec 7;66(4):411–9. doi: 10.5194/aab-66-411-2023 (PMC10776882; doi:10.5194/aab-66-411-2023)

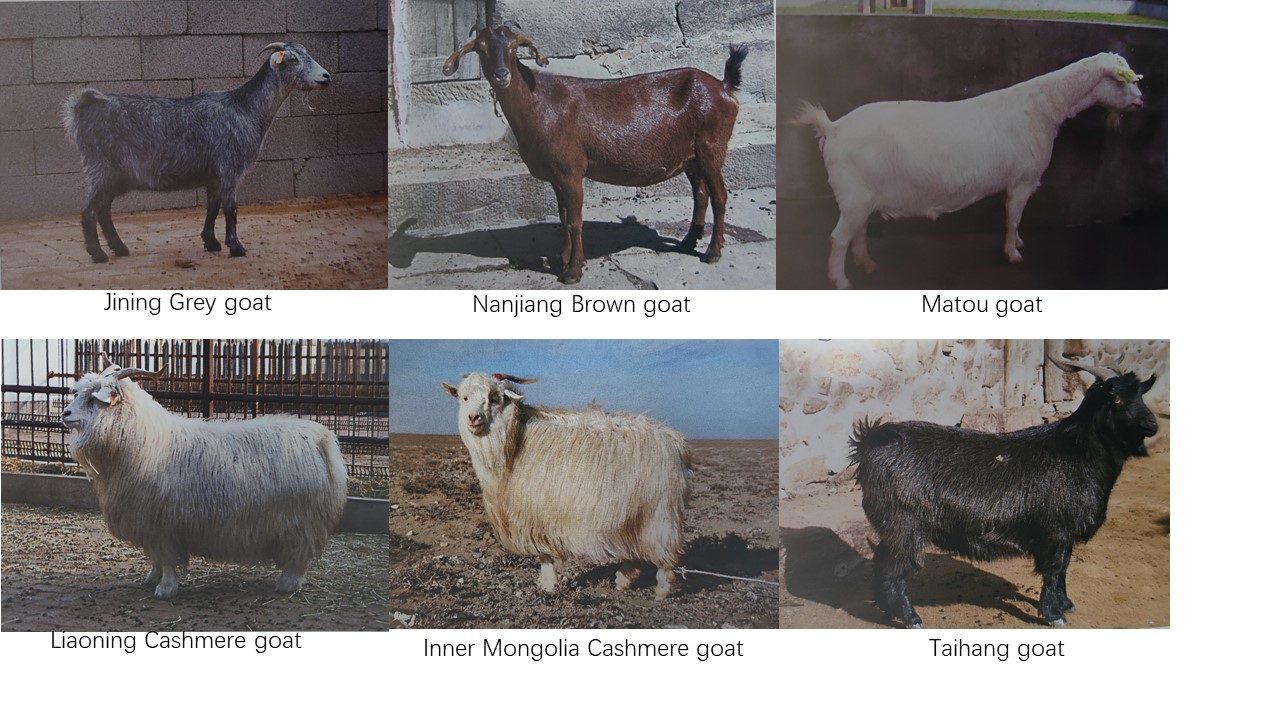

Supplement: The supplement related to this article is available online at: https://doi.org/10.5194/aab-66-411-2023-supplement. [file aab-66-411-supplement.zip › Figure S1. Photos for different breeds.jpg]
